# Genetic variants and serum biomarkers of *CXCL8*,* MAP3K7*,* LTA/TNF*,* EXOC3L1*,* PROCR*, and *TRAF2* in Age-Related macular degeneration: associations with disease risk and therapeutic response

**DOI:** 10.1038/s41598-026-42838-9

**Published:** 2026-03-17

**Authors:** Dzastina Cebatoriene, Alvita Vilkeviciute, Monika Duseikaite-Vidike, Enrika Pileckaite, Akvile Bruzaite, Loresa Kriauciuniene, Dalia Zaliuniene, Rasa Liutkeviciene

**Affiliations:** 1 Medical Academy, Lithuanian University of Health Sciences, A. Mickeviciaus St. 9, Kaunas, LT-44307 Lithuania; 2 Neuroscience Institute, Medical Academy, Lithuanian University of Health Sciences, Eiveniu St. 2, Kaunas, LT-50161 Lithuania; 3 Department of Ophthalmology, Medical Academy, Lithuanian University of Health Sciences, Eiveniu St. 2, Kaunas, LT-50161 Lithuania

**Keywords:** AMD, Anti-VEGF therapy, Protein levels, SNV analysis, Biomarkers, Diseases, Genetics, Medical research

## Abstract

Since age-related macular degeneration (AMD) is the leading cause of irreversible central vision loss in the aging population, it is a significant global health concern. Although anti-vascular endothelial growth factor (anti-VEGF) treatments are effective, not all patients respond to them fully. This study focuses on key single-nucleotide variants in the *CXCL8* (rs2227306), *MAP3K7* (rs157432), *LTA/TNF* (rs2229094), *EXOC3L1* (rs868213), *PROCR* (rs867186), *TRAF2* (rs10781522), and serum levels of these genes in AMD development and treatment response. It examines the genetic factors associated with susceptibility to AMD and how they influence response to therapy. The study investigates the relationships between specific genetic variations, serum protein levels, and both exudative and early AMD, as well as responses to anti-VEGF treatment. These findings may help guide risk assessment and personalized AMD therapies.

**Supplementary Information:**

The online version contains supplementary material available at 10.1038/s41598-026-42838-9.

## Introduction

Age-related macular degeneration (AMD) is a progressive neurodegenerative disease of the central retina and a leading cause of irreversible central vision loss in older adults worldwide^1,2^. The global burden of AMD is expected to increase substantially with population aging, particularly in Europe and North America^3^. Although AMD rarely leads to complete blindness, loss of central vision severely affects reading ability, facial recognition, driving, and overall quality of life^4^.

AMD is a multifactorial disease arising from a complex interplay between genetic susceptibility, aging, environmental exposures, oxidative stress, and chronic inflammation^5,6^. These factors contribute to retinal pigment epithelium (RPE) dysfunction, photoreceptor degeneration, drusen formation, and, in the neovascular subtype, choroidal neovascularization (CNV)^7,8^. Genome-wide association studies (GWAS) have robustly identified risk variants in complement pathway genes such as *CFH*, *C3*, and *ARMS2*, establishing a strong genetic component in AMD pathogenesis^9,10^. Moreover, large-scale genetic studies have demonstrated stage-specific differences in genetic associations, including the differential impact of both common and rare variants across AMD stages^9^.

However, these loci do not fully explain disease heritability, suggesting that additional genetic contributors remain to be elucidated.

Increasing evidence indicates that genes involved in inflammatory signaling, immune regulation, intracellular trafficking, and vascular homeostasis may modulate AMD susceptibility and progression. The present study focused on CXCL8 (IL-8), MAP3K7, LTA, EXOC3L1, and PROCR, selected based on a combination of (i) prior genetic association evidence or biomarker data in AMD or related retinal conditions, (ii) functional involvement in pathways repeatedly implicated in AMD pathophysiology (inflammation, angiogenesis, oxidative stress response, and endothelial function), and (iii) limited or inconsistent prior investigation in AMD populations, highlighting their potential as underexplored candidates.

Among these, *CXCL8* has the strongest prior support. The rs2227306 polymorphism, located in the promoter region of *CXCL8*, has been associated with increased AMD risk, particularly neovascular AMD, in several populations^11,12^. Elevated IL-8 levels have been detected in the aqueous humor and blood of patients with neovascular AMD, supporting its relevance as both a genetic and biomarker candidate^13^. IL-8 is of particular interest because it promotes angiogenesis through VEGF-independent mechanisms, which may contribute to therapeutic resistance in some patients.

*MAP3K7*, encoding transforming growth factor-β–activated kinase 1 (TAK1), was selected due to its central role in NF-κB and MAPK signaling pathways activated by oxidative stress and inflammatory stimuli—core processes in AMD. Although direct associations between *MAP3K7* variants and AMD remain limited, pathway enrichment and systems biology analyses consistently implicate MAPK signaling in advanced AMD, supporting *MAP3K7* as a biologically plausible candidate^14,15^.

*LTA* was included as a representative of TNF-family inflammatory signaling. While direct associations between *LTA* variants and AMD have been inconsistent, TNF-mediated inflammation has been repeatedly implicated in retinal degeneration and CNV. Moreover, the genomic proximity of *LTA* to immune-related loci raises the possibility of indirect genetic effects through linkage disequilibrium^16,17^.

*EXOC3L1* was selected as an exploratory candidate due to its role in vesicular trafficking and exocytosis, processes essential for RPE function, autophagy, and photoreceptor outer segment clearance. Disruption of exocyst components has been shown to impair RPE homeostasis and promote retinal degeneration in experimental models^18–20^, suggesting a potential indirect contribution to AMD pathogenesis.

Finally, *PROCR*, encoding the endothelial protein C receptor (EPCR), was chosen based on emerging evidence linking EPCR signaling to pathological retinal neovascularization. Experimental studies have demonstrated increased EPCR expression in retinal neovascular lesions and reduced abnormal vessel growth following EPCR inhibition^21^, indicating a possible role in neovascular AMD despite limited genetic data to date.

Together, these genes represent established and emerging candidates spanning complementary but distinct biological pathways relevant to AMD. By evaluating their genetic and biomarker associations, this study aims to broaden the understanding of non-complement-mediated contributors to AMD susceptibility and progression.

## Materials and methods

### Ethics

The Lithuanian University of Health Sciences’ Kaunas Regional Biomedical Research Ethics Committee approved this study, which complied with the Declaration of Helsinki’s principles (approval number BE-2-/48). All participants provided their informed consent.

### Study design and study group formation

Between 2014 and 2024, the subjects were admitted to the Hospital of the Lithuanian University of Health Sciences’ Ophthalmology Department, where participants received a comprehensive ophthalmic assessment, including best-corrected visual acuity (BCVA) measured with the Early Treatment Diabetic Retinopathy Study (ETDRS) chart, fundus photography, structural optical coherence tomography (OCT; Triton SS-OCT, Topcon, Tokyo, Japan) for central macular thickness (CMT) evaluation, and OCT angiography (OCT-A); fluorescein angiography was performed when clinically indicated.

AMD was categorized according to the American Academy of Ophthalmology criteria. Early AMD was defined by the presence of numerous small (< 63 μm, hard) or intermediate (≥ 63 μm and < 125 μm, soft) drusen. Intermediate AMD was characterized by extensive small or intermediate drusen or the presence of any large drusen (≥ 125 μm). Advanced AMD was defined by geographic atrophy or choroidal neovascularization, including associated findings such as subretinal or sub-RPE hemorrhage or fluid and subretinal fibrosis. Medical records and general practitioner examinations were used to gather health information and specifics about additional medical conditions. Before being included in the study, each subject gave their informed consent.

Participants were subsequently divided into two groups: an AMD group and a control group. Patients assigned to the AMD group were aged ≥ 55 years and had a verified diagnosis of early or exudative AMD, with available BCVA and CMT measurements and documented follow-up during anti–vascular endothelial growth factor (anti-VEGF) therapy when applicable. Control subjects were aged ≥ 18 years, had undergone cataract surgery, and had no ocular comorbidities.

Exclusion criteria for both groups included the presence of non-AMD ocular diseases, significant systemic illnesses (e.g., diabetes mellitus, malignancy, stroke, or organ transplantation), ungradable fundus photographs, and the use of antiepileptic or sedative medications. AMD patients were evaluated and followed by an ophthalmologist according to predefined clinical protocols. Detailed inclusion and exclusion criteria for AMD patients and control subjects have been described in our previous paper^22^. All analyses were performed per eye. In cases where both eyes were eligible, only one eye per participant was included to avoid inter-eye correlation, with the study eye selected based on disease presence and severity.

The study comprised 946 subjects categorized into a control group (*n* = 333), early AMD (*n* = 284), and exudative AMD (*n* = 329) groups. The control group was adjusted concerning gender to match the early and exudative AMD groups (*p* = 0.275 and *p* = 0.974, respectively). Median age (IQR) was 72 (11) years in the control group, 74 (13) years in the early AMD group, and 77 (9.75) years in the exudative AMD group; age differed significantly between the control and exudative AMD groups (*p* < 0.001) but not between the control and early AMD groups (*p* = 0.065). necessitating further analysis adjusted by age. Demographic information for all study subjects is detailed in Table 1, providing a comprehensive overview of the characteristics within all groups.

**Table 1:** Demographic data of the study.

Characteristics		Groups			*p*-value
		Control	Early AMD	Exudative AMD	
Gender	Females, n (%)	216 (64.9)	196 (69.0)	213 (64.7)	0.275* 0.974**
	Males, n (%)	117 (35.1)	88 (31.0)	116 (35.3)	
Age, Median (IQR)		72 (11)	74 (13)	77 (9.75)	0.065* < 0.001**

*p* - significance level; IQR - interquartile range; *early AMD vs. control group; **exudative AMD vs. control group.

### Exudative AMD response to anti-VEGF injection treatment

Patients with exudative AMD, which is characterised by exudative or hemorrhagic macular characteristics, were used to test the effectiveness of anti-VEGF medications (Ranibizumab, Aflibercept, and Bevacizumab). Three to six months after treatment, patients who had never received intravitreal anti-VEGF injections before were monitored. Visual acuity (VA) and central retinal thickness (CRT) were assessed before therapy as well as three and six months later. Based on clinical optical coherence tomography (OCT) and best corrected visual acuity (BCVA) results, patients were classified as either responders or non-responders. In a prior article, specific procedures and standards for assessing responses were detailed, including definitions of structural alterations, declines in visual acuity, and patient classification^22^.

### DNA extraction from peripheral venous blood and genotyping

Blood for Deoxyribonucleic acid (DNA) extraction was collected in EDTA tubes. Genomic DNA was extracted using the DNA salting-out method from peripheral blood (white blood cells). The concentrations and purity indexes of DNA in each blood sample were evaluated by UV spectrophotometry (Agilent Technologies, Cary 60 UV– Vis) as the ratio absorbance 260/280 nm. DNA extraction and genotyping of selected single-nucleotide variants (SNVs) - *CXCL8* rs2227306, *MAP3K7* rs157432, *TNF/LTA* rs2229094, *EXOC3L1* rs868213, *PROCR* rs867186, and *TRAF2* rs10781522—were performed at the Laboratory of Ophthalmology, Neuroscience Institute, Lithuanian University of Health Sciences, using predesigned TaqMan™ Genotyping assays (Thermo Fisher Scientific, Pleasanton, CA, USA) following the manufacturer’s recommendations. The genotyping was conducted using the real‐time polymerase chain reaction (RT‐PCR) method according to the manufacturer’s recommendations using a Step One Plus RT‐PCR system (Applied Biosystems, Chicago, IL, USA).

### SNV selection

Many researchers have looked at AMD from a whole-genome perspective. Whole exome sequencing (WES), which is carried out on genomic regions of the genome that code for proteins, is a powerful method for identifying genetic variants that may be connected to AMD.

Our study’s SNVs were carefully chosen because of their diverse and varied connections to disease processes. We looked closely at previous studies on these variations and how they relate to different diseases. Following a thorough analysis, we determined which SNVs were the most important to investigate in relation to AMD, its stages, and possible therapies (Table 2).

**Table 2:** SNV position and selection.

SNV	Position	Associations
*CXCL8* rs2227306	Intron variant	rs2227306 and specific *CXCL8* haplotypes have been linked to increase AMD risk in multiple populations^12^.
*MAP3K7* rs157432	Intron variant	*MAP3K7* gene encodes TGF-β-activated kinase 1 (TAK1), which plays a vital role in innate and adaptive immunity by regulating inflammatory responses and regulating cell differentiation, cell survival, and apoptosis^23^.
*TNF/LTA* rs2229094	Upstream variant	The rs2229094 variant in the *LTA* and Proliferative Vitreoretinopathy (PVR), a condition marked by inflammation and retinal scarring. This SNV affects the signal peptide region of lymphotoxin-α, potentially altering inflammatory signaling^24^.
*EXOC3L1* rs868213	Intron variant	The rs868213 variant was strongly associated with HDL cholesterol levels in African Americans, linking it indirectly to vascular health and inflammation^25^.
*PROCR* rs867186	Intron variant	rs867186 affects the risk of thrombotic events, exhibiting a complex pattern of associations with both venous and arterial diseases. By modulating protein C levels and promoting the shedding of EPCR from the endothelial membrane, it plays a key role in the anticoagulant pathway and influences related clinical phenotypes^26–28^.
*TRAF2* rs10781522	Intron variant	rs10781522 is implicated in the regulation of gene expression and is associated with disease susceptibility and severity across multiple conditions, particularly autoimmune diseases such as pemphigus foliaceus (PF), where it has a protective effect^29–31^. Its role is likely linked to TRAF2’s function in immune signaling and cell fate determination. Notably, in a mouse model of glaucoma, overexpression of the tetraspanin CD82 protects retinal ganglion cells by increasing TRAF2 levels, which are associated with disease, and by activating the mTORC1 pathway to maintain axonal transport under elevated intraocular pressure^32^.

### Serum protein concentration measurement

The serum was prepared by centrifuging blood that had been extracted from peripheral veins and left to incubate for 30 min at room temperature. Serum samples were collected using clot activator Vacuum Lind-Vac tubes. The serum was carefully removed from the cell pellet after centrifugation, then placed into 2 mL containers and kept at −80 °C until analysis. In accordance with the manufacturer’s instructions, the serum levels of CXCL8, MAP3K7, TNF/LTA, EXOC3L1, PROCR, and TRAF2 were assessed in both AMD patients and control subjects.

Serum levels of the control, early, and exudative AMD patient groups were measured using the enzymatic immunoassay (ELISA) based on the conventional sandwich ELISA technique. The measurements were taken according to the manufacturer’s specifications. The optical density at 450 nm was measured using a microplate reader (Multiskan FC microplate photometer, Thermo Scientific, Waltham, MA, USA). The CXCL8, MAP3K7, TNF/LTA, EXOC3L1, PROCR, and TRAF2 serum levels were determined using the standard curve. More information is presented in Supplementary material 1, Table 1.

### Statistical analysis

Software SPSS/W 30.0 (Statistical Package for the Social Sciences for Windows, Inc., Chicago, IL, USA) was used to do the statistical analysis. The Shapiro-Wilk test was used to determine whether the continuous variables (age, VA, and CRT) were normal. The non-parametric Mann-Whitney U test was used to compare continuous variables that did not fit the normal distribution model. These variables were expressed as median with interquartile range (IQR). The VA and CRT differences before and after therapy were compared using the nonparametric Wilcoxon signed-rank test. The threshold for statistical significance was set at *p* < 0.05. Using Pearson’s chi-squared test (χ2), categorical data (gender and genotype distributions) were compared between groups and displayed as absolute numbers with percentages in parentheses. The influence of gene variants on early and exudative AMD was assessed using binomial logistic regression analysis. The findings for the logistic regression analysis were shown as odds ratios (OR) with a 95% CI (confidence interval). Age-adjusted odds ratios (OR) and 95% CIs for the exudative AMD groups were used to display the results. Codominant, dominant, recessive, and overdominant genetic models were used, while the additive model evaluated how each minor allele affected AMD. Multiple genetic inheritance models were explored because the true mode of action of the studied variants in AMD is unknown. The codominant and additive models were considered primary for interpretation, as they provide genotype-specific and per-allele risk estimates, respectively, while dominant, recessive, and overdominant models were included for exploratory purposes.We used the significance level (p) criteria to test statistical hypotheses, applied the Bonferroni adjustment to the analysis, and decided that a difference was statistically significant when the p-value was less than 0.008.

## Results

### Hardy–weinberg equilibrium analysis

Baseline demographic characteristics of the study population are summarized in Table 1Hardy–Weinberg equilibrium (HWE) was evaluated separately in controls, early AMD, and exudative AMD groups. It confirmed that the genotype frequencies of *CXCL8* rs2227306, *MAP3K7* rs157432, *TNF/LTA* rs2229094, *PROCR* rs867186, and *TRAF2* rs10781522 showed no significant deviation from HWE in the patient and control groups (*p* > 0.05). Only the EXOC3L1 rs868213 variant deviated from HWE in the control and early AMD groups, but not in the exudative AMD group (*p* < 0.05). Notably, genotype distributions demonstrated a consistent decrease in the frequency of the GG genotype from controls to early AMD and further to exudative AMD, suggesting genotype-dependent selection related to disease presence or progression. Given the biologically plausible pattern, comparable allele frequencies, and absence of indications of genotyping error, the variant was retained for association analyses, and results were interpreted with appropriate caution. Results presented in the Table 3.

**Table 3:** Hardy–Weinberg equilibrium evaluation in study groups.

SNV/Group		Observed frequencies					Expected frequencies			HWE *p*
CXCL8 rs2227306	*n*	CC	CT	TT	C	T	CC	CT	TT	
Early AMD	284	68	153	63	0.509	0.491	73.6	142.0	68.4	0.189
Exudative AMD	329	80	172	77	0.505	0.495	83.9	164.3	80.8	0.407
Controls	333	102	159	72	0.545	0.455	98.9	165.5	68.6	0.497
***MAP3K7*** **rs157432**	**n**	**CC**	**CT**	**TT**	**C**	**T**	**CC**	**CT**	**TT**	**HWE p**
Early AMD	284	173	95	16	0.776	0.224	171.2	98.7	14.1	0.538
Exudative AMD	329	186	127	16	0.758	0.242	188.8	120.8	19.4	0.334
Controls	333	196	120	17	0.789	0.211	207.6	110.7	14.7	0.804
***TNF/LTA*** **rs2229094**	**n**	**TT**	**TC**	**CC**	**T**	**C**	**TT**	**TC**	**CC**	**HWE p**
Early AMD	284	168	97	19	0.762	0.238	164.7	102.8	16.5	0.333
Exudative AMD	329	182	126	21	0.745	0.255	182.7	124.7	21.6	0.897
Controls	333	197	124	12	0.778	0.222	201.7	114.9	16.4	0.159
***EXOC3L1*** **rs868213**	**n**	**AA**	**AG**	**GG**	**A**	**G**	**AA**	**AG**	**GG**	**HWE p**
Early AMD	284	252	27	5	0.935	0.065	248.5	34.7	**1.2**	**0.003**
Exudative AMD	329	300	28	1	0.954	0.046	300.2	27.8	0.8	0.689
Controls	333	252	61	20	0.848	0.152	239.6	85.7	7.7	**< 0.001**
***PROCR*** **rs867186**	**n**	**AA**	**AG**	**GG**	**A**	**G**	**AA**	**AG**	**GG**	**HWE p**
Early AMD	284	221	60	3	0.884	0.116	221.6	58.3	3.8	0.630
Exudative AMD	329	247	77	5	0.868	0.132	247.8	75.4	5.7	0.718
Controls	333	270	60	3	0.901	0.099	270.5	59.3	3.2	0.868
***TRAF2*** **rs10781522**	**n**	**AA**	**AG**	**GG**	**A**	**G**	**AA**	**AG**	**GG**	**HWE p**
Early AMD	284	140	118	26	0.701	0.299	139.5	118.7	25.8	0.874
Exudative AMD	329	143	153	33	0.667	0.333	146.4	146.1	36.5	0.392
Controls	333	147	148	38	0.664	0.336	147.0	148.7	37.3	0.935

### SNVs associations with early and exudative AMD occurrence

Genotype and allele distributions were analyzed separately for early and exudative AMD to account for biological and clinical heterogeneity between disease stages. A combined AMD-versus-control analysis was not prioritized, as pooling stages could mask stage-specific genetic effects. After analyzing the genotypes and alleles of *CXCL8* rs2227306, *MAP3K7* rs157432, *TNF/LTA* rs2229094, *EXOC3L1* rs868213, *PROCR* rs867186, and *TRAF2* rs10781522, we found that the distribution of *EXOC3L1* rs868213 AA, AG, and GG genotypes is statistically significantly different in both early and exudative AMD groups compared with the control (88.7%, 9.5%, and 1.8% vs. 75.7%, 18.3%, and 6.0%, *p* < 0.001; 91.2%, 8.5%, and 0.3% vs. 75.7%, 18.3%, and 6.0%, *p* < 0.001, respectively). The G allele of rs868213 was statistically significantly less frequent in patients with early and exudative AMD compared to the control group (6.5% vs. 15.2%, *p* < 0.001; 4.6% vs. 15.2%, *p* < 0.001, respectively). No statistically significant differences were found between the distribution of genotypes and alleles of *CXCL8* rs2227306, *MAP3K7* rs157432, *TNF/LTA* rs2229094, *PROCR* rs867186, and *TRAF2* rs10781522 in patients with early and exudative AMD and the control group (Table 4).

**Table 4:** Distributions of *CXCL8*, *MAP3K7*, *TNF/LTA*, *EXOC3L1*, *PROCR*, and *TRAF2* SNVs genotypes and alleles in early, exudative AMD and control groups.

Gene/marker	Genotype/ allele	Group			*p*-value*	*p*-value**
		Early AMD* (*n* = 284) *n* (%)	Exudative AMD** (*n* = 329) *n* (%)	Control (*n* = 333) *n* (%)		
*CXCL8* rs2227306	CC CT TT C T	68 (23.9) 153 (53.9) 63 (22.2) 289 (50.9) 279 (49.1)	80 (24.3) 172 (52.3) 77 (23.4) 332 (50.5) 326 (49.5)	102 (30.6) 159 (47.7) 72 (21.6) 363 (54.5) 303 (45.5)	0.161 0.204	0.191; 0.140
*MAP3K7* rs157432	CC CT TT C T	173 (60.9) 95 (33.5) 16 (5.6) 441 (77.6) 127 (22.4)	186 (56.5) 127 (38.6) 16 (4.9) 499 (75.8) 159 (24.2)	196 (58.9) 120 (36.0) 17 (5.1) 512 (78.9) 154 (21.1)	0.786 0.750	0.792; 0.656
*TNF/LTA* rs2229094	TT TC CC T C	168 (59.2) 97 (34.2) 19 (6.7) 433 (76.2) 135 (23.8)	182 (55.3) 126 (38.3) 21 (6.4) 490 (74.5) 168 (25.5)	197 (59.2) 124 (37.2) 12 (3.6) 518 (77.8) 148 (22.2)	0.191 0.520	0.219; 0.158
*EXOC3L1* rs868213	AA AG GG A G	252 (88.7) 27 (9.5) 5 (1.8) 531 (93.5) 37 (6.5)	300 (91.2) 28 (8.5) 1 (0.3) 628 (95.4) 30 (4.6)	252 (75.7) 61 (18.3) 20 (6.0) 565 (84.8) 101 (15.2)	**< 0.001** **< 0.001**	**< 0.001;** **< 0.001**
*PROCR* rs867186	AA AG GG A G	221 (77.8) 60 (21.1) 3 (1.1) 502 (88.4) 66 (11.6)	247 (75.1) 77 (23.4) 5 (1.5) 571 (86.8) 87 (13.2)	270 (81.1) 60 (18.0) 3 (0.9) 600 (90.1) 66 (9.9)	0.605 0.333	0.165; 0.059
*TRAF2* rs10781522	AA AG GG A G	140 (49.3) 118 (41.5) 26 (9.2) 398 (70.1) 170 (29.9)	143 (43.5) 153 (46.5) 33 (10.0) 439 (66.7) 219 (33.3)	147 (44.1) 148 (44.4) 38 (11.4) 442 (66.4) 224 (33.6)	0.382 0.164	0.792; 0.892

p- significance level, significance level *p* = 0.008.

* Early AMD vs. control group,.

** exudative AMD vs. control group.

After analyzing the influence of early AMD occurrence, we found that the *EXOC3L1* rs868213 was associated with 2.6-fold and 4-fold decreased odds of early AMD development under the codominant model (OR = 0.443; 95% CI: 0.272–0.719; *p* < 0.001; OR = 0.250; 95% CI: 0.092–0.676; *p* = 0.006, respectively). Also, GG + AG genotypes compared with the AA genotype, and AG genotype compared with AA + GG genotypes had a 2.5-fold, and 2.1-fold decreased odds of developing early AMD under the dominant, and overdominant genetic models, respectively (OR = 0.395; CI: 0.253–0.616; *p* < 0.001and OR = 0.468; CI: 0.289–0.760; *p* = 0.002, respectively). Finally, each G allele 2.1-fold decreased the odds of early AMD under the additive model (OR = 0.469; 95% CI: 0.326–0.674; *p* < 0.001) (Table 5).

**Table 5:** Binomial logistic regression analysis of *CXCL8*, *MAP3K7*, *TNF/LTA*, *EXOC3L1*, *PROCR*, and *TRAF2* in the control and patients with early AMD groups.

Model	Genotype/allele	OR (95% CI)	*p*-value	AIC
*CXCL8* rs2227306				
Codominant	CT vs. CC TT vs. CC	1.443 (0.988–2.108) 1.312 (0.831–2.072)	0.058 0.243	851.782
Dominant	TT + CT vs. CC	1.403 (0.980–2.007)	0.064	849.994
Recessive	TT vs. CC + CT	1.033 (0.705–1.515)	0.866	853.420
Overdominant	CT vs. TT + CC	1.278 (0.931–1.755)	0.130	851.146
Additive	T	1.159 (0.924–1.454)	0.201	851.807
*MAP3K7* rs157432				
Codominant	CT vs. CC TT vs. CC	0.897 (0.640–1.258) 1.066 (0.523–2.175)	0.528 0.860	854.965
Dominant	TT + CT vs. CC	0.918 (0.664–1.268)	0.604	853.178
Recessive	TT vs. CC + CT	1.110 (0.550–2.239)	0.771	853.364
Overdominant	CT vs. TT + CC	0.892 (0.640–1.245)	0.502	852.996
Additive	T	0.958 (0.734–1.250)	0.751	853.347
*TNF/LTA* rs2229094				
Codominant	TC vs. TT CC vs. TT	0.917 (0.655–1.284) 1.857 (0.874–3.936)	0.615 0.107	852.136
Dominant	CC + TC vs. TT	1.000 (0.725–1.380)	0.999	853.448
Recessive	CC vs. TT + TC	1.918 (0.914–4.023)	0.085	850.390
Overdominant	TC vs. TT + CC	0.874 (0.628–1.217)	0.426	852.814
Additive	C	1.093 (0.836–1.428)	0.517	853.029
*EXOC3L1* rs868213				
Codominant	AG vs. AA GG vs. AA	0.443 (0.272–0.719) 0.250 (0.092–0.676)	**< 0.001** **0.006**	836.222
Dominant	GG + AG vs. AA	0.395 (0.253–0.616)	**< 0.001**	835.374
Recessive	GG vs. AA + AG	0.280 (0.104–0.757)	0.012	845.753
Overdominant	AG vs. AA + GG	0.468 (0.289–0.760)	**0.002**	843.434
Additive	G	0.469 (0.326–0.674)	**< 0.001**	834.347
*PROCR* rs867186				
Codominant	AG vs. AA GG vs. AA	1.222 (0.819–1.822) 1.222 (0.244–6.113)	0.326 0.807	854.445
Dominant	GG + AG vs. AA	1.222 (0.826–1.808)	0.317	852.445
Recessive	GG vs. AA + AG	1.174 (0.235–5.865)	0.845	853.410
Overdominant	AG vs. AA + GG	1.219 (0.818–1.816)	0.331	852.505
Additive	G	1.199 (0.833–1.727)	0.329	852.495
*TRAF2* rs10781522				
Codominant	AG vs. AA GG vs. AA	0.837 (0.599–1.170) 0.718 (0.415–1.245)	0.298 0.239	853.519
Dominant	GG + AG vs. AA	0.813 (0.592–1.117)	0.201	851.813
Recessive	GG vs. AA + AG	0.782 (0.462–1.324)	0.360	852.603
Overdominant	AG vs. AA + GG	0.889 (0.645–1.224)	0.469	852.924
Additive	G	0.844 (0.663–1.073)	0.166	851.523

OR - odds ratio; CI - confidence interval; *p* - significance level; AIC - Akaike information criteria.

Binary logistic regression analysis revealed that *CXCL8* rs2227306 CT vs. CC and TT + CT vs. CC were associated with about 1.5-fold increased odds of exudative AMD occurrence under the codominant and dominant genetic models (OR = 1.526; CI: 1.038–2.242; *p* = 0.031; OR = 1.525; CI: 1.060–2.194; *p* = 0.023, respectively). When we applied Bonferroni corrected significance threshold, these results did not reach statistical significance.

*EXOC3L1* rs868213 AG genotype compared with the AA genotype and GG genotype compared with the AA genotype decreased the possibility of exudative AMD by 2.6-fold and 17.0-fold (OR = 0.387, 95% CI: 0.235–0.637, *p* < 0.001, OR = 0.059, 95% CI: 0.008–0.457, *p* = 0.007, respectively) under the codominant model. Furthermore, GG + AG genotypes compared with the AA genotype, and AG genotype compared with AA + GG genotypes had a 3.1-fold, and 2.4-fold decreased odds of developing exudative AMD under the dominant, and over-dominant genetic models, respectively (OR = 0.321; CI: 0.200–0.517.200.517; *p* < 0.001,and OR = 0.409; CI: 0.248–0.673; *p* < 0.001, respectively). Also, each G allele decreased these odds by 2.9-fold under the additive model (OR = 0.347, 95% CI: 0.226–0.532, *p* < 0.001) (Table 6).

**Table 6:** Binomial logistic regression analysis of *CXCL8*, *MAP3K7*, *TNF/LTA*, *EXOC3L1*, *PROCR*, and *TRAF2* in the control and patients with exudative AMD groups.

Model	Genotype/allele	OR (95% CI)	*p*-value	AIC
*CXCL8* rs2227306				
Codominant	CT vs. CC TT vs. CC	1.526 (1.038–2.242) 1.524 (0.963–2.410)	0.031 0.072	854.582
Dominant	TT + CT vs. CC	1.525 (1.060–2.194)	0.023	852.582
Recessive	TT vs. CC + CT	1.155 (0.789–1.692)	0.459	857.249
Overdominant	CT vs. CC + TT	1.257 (0.912–1.731)	0.162	855.839
Additive	T	1.245 (0.990–1.565)	0.061	854.255
*MAP3K7* rs157432				
Codominant	CT vs. CC TT vs. CC	1.049 (0.750–1.466) 0.868 (0.409–1.843)	0.781 0.713	859.542
Dominant	TT + CT vs. CC	1.026 (0.743–1.418)	0.876	857.775
Recessive	TT vs. CC + CT	0.852 (0.406–1.787)	0.671	857.619
Overdominant	CT vs. CC + TT	1.060 (0.762–1.475)	0.728	857.678
Additive	T	0.997 (0.760–1.308)	0.981	857.799
*TNF/LTA* rs2229094				
Codominant	TC vs. TT CC vs. TT	1.086 (0.777–1.518) 1.836 (0.843–3.995)	0.628 0.126	857.337
Dominant	CC + TC vs. TT	1.152 (0.834–1.591)	0.392	857.066
Recessive	CC vs. TT + TC TC vs. TT + CC	1.777 (0.826–3.823)	0.142	855.571
Overdominant	TC vs. TT + CC CC vs. TC + TT	1.037 (0.746–1.441)	0.830	857.753
Additive	C	1.191 (0.907–1.564)	0.208	856.206
*EXOC3L1* rs868213				
Codominant	AG vs. AA GG vs. AA	0.387 (0.235–0.637) 0.059 (0.008–0.457)	**< 0.001** **0.007**	830.917
Dominant	GG + AG vs. AA	0.321 (0.200–0.517.200.517)	**< 0.001**	833.856
Recessive	GG vs. AA + AG	0.067 (0.009–0.518)	0.010	843.750
Overdominant	AG vs. AA + GG	0.409 (0.248–0.673)	**< 0.001**	844.701
Additive	G	0.347 (0.226–0.532)	**< 0.001**	829.694
*PROCR* rs867186				
Codominant	AG vs. AA GG vs. AA	1.319 (0.888–1.958) 1.873 (0.428–8.191)	0.170 0.405	857.317
Dominant	GG + AG vs. AA	1.345 (0.914–1.980)	0.133	855.530
Recessive	GG vs. AA + AG	1.768 (0.405–7.716)	0.449	857.206
Overdominant	AG vs. AA + GG	1.306 (0.880–1.938)	0.185	856.037
Additive	G	1.329 (0.931–1.897)	0.117	855.325
*TRAF2* rs10781522				
Codominant	AG vs. AA GG vs. AA	1.086 (0.775–1.522) 0.922 (0.531–1.599)	0.633 0.771	859.356
Dominant	GG + AG vs. AA	1.053 (0.763–1.453)	0.752	857.699
Recessive	GG vs. AA + AG	0.884 (0.524–1.491)	0.643	857.584
Overdominant	AG vs. AA + GG	1.103 (0.800–1.520)	0.549	857.440
Additive	G	1.003 (0.786–1.279)	0.982	857.799

OR - odds ratio; CI - confidence interval; *p* - significance level; AIC - Akaike information criteria.

### SNVs associations with early and exudative AMD occurrence by gender

The findings of genotypes and alleles in early, exudative AMD and control groups between different gender distributions suggest that, in females the distribution of CC, CT, and TT genotypes of *CXCL8* rs2227306 is statistically significantly different in early AMD patients compared to the control group (20.4%, 59.7% and 19.9% vs. 29.2%, 47.2% and 23.6%, *p* = 0.033). Unfortunately, these results did not survive strict Bonferroni correction.The distribution of *EXOC3L1* rs868213 AA, AG, and GG genotypes is statistically significantly different in both early and exudative AMD females compared with the healthy females (89.3%, 8.7%, and 2.0% vs. 76.4%, 15.7%, and 7.9%, *p* = 0.001; 91.5%, 8.0%, and 0.5% vs. 76.4%, 15.7%, and 7.9%, *p* < 0.001, respectively). The G allele of rs868213 was statistically significantly less frequent in females with early and exudative AMD compared to the control group women (6.4% vs. 15.7%, *p* < 0.001; 4.5% vs. 15.7%, *p* < 0.001, respectively) (Table 7).

**Table 7:** Distributions of *CXCL8*, *MAP3K7*, *TNF/LTA*, *EXOC3L1*, *PROCR*, and *TRAF2* SNVs genotypes and alleles in early, exudative AMD and control females and males.

Gene/marker	Genotype/allele	Group						*p*-value*	*p*-value**	*p*-value***	*p*-value****
		Early AMD females*	Early AMD males***	Exudative AMD females**	Exudative AMD males****	Control females	Control				
		(n=196)	(n=88)	(n=213)	(n=116)	(n=216)	males (n=117)				
		n (%)	n (%)	n (%)	n (%)	n (%)	n (%)				
CXCL8 rs2227306				.				0.033	0.394	0.259	0.438
	CC	40 (20.4)	28 (31.8)	50 (23.5)	30 (25.9)	63 (29.2)	39 (33.3)				
	CT	117 (59.7)	36 (40.9)	111 (52.1)	61 (52.6)	102 (47.2)	57 (48.7)				
	TT	39 (19.9)	24 (27.3)	52 (24.4)	25 (21.6)	51 (23.6)	21 (17.9)				
				.				0.469	0.341	0.275	0.23
	C	197 (50.3)	92 (52.3)	211 (49.5)	121 (52.2)	228 (52.8)	135 (57.7)				
	T	195 (49.7)	84 (47.7)	215 (50.5)	111 (47.8)	204 (47.2)	99 (42.3)				
MAP3K7 rs157432								0.472	0.767	0.423	0.955
	CC	119 (60.7)	54 (61.4)	120 (56.3)	66 (56.9)	129 (59.7)	67 (57.3)				
	CT	63 (32.1)	32 (36.4)	83 (39.0)	44 (37.9)	77 (35.6)	43 (36.8)				
	TT	14 (7.1)	2 (2.3)	10 (4.7)	6 (5.2)	10 (4.6)	7 (6.0)				
								0.795	0.55	0.35	0.956
	C	301 (76.8)	140 (79.5)	323 (75.8)	176 (75.9)	335 (77.5)	177 (75.6)				
	T	91 (23.2)	36 (20.5)	103 (24.2)	56 (24.1)	97 (22.5)	57 (24.4)				
TNF/LTArs2229094								0.243	0.157	0.457	0.925
	TT	119 (60.7)	49 (55.7)	110 (51.6)	72 (62.1)	125 (57.9)	72 (61.5)				
	TC	64 (32.7)	33 (37.5)	87 (40.8)	39 (33.6)	83 (38.4)	41 (35.0)				
	CC	13 (6.6)	6 (6.8)	16 (7.5)	5 (4.3)	8 (3.7)	4 (3.4)				
								0.988	0.091	0.27	0.962
	T	302 (77.0)	131 (74.4)	307 (72.1)	183 (78.9)	333 (77.1)	185 (79.1)				
	C	90 (23.0)	45 (25.6)	119 (27.9)	49 (21.1)	99 (22.9)	49 (20.9)				
EXOC3L1 rs868213								0.001	<0.001	0.066	0.003
	AA	175 (89.3)	77 (87.5)	195 (91.5)	105 (90.5)	165 (76.4)	87 (74.4)				
	AG	17 (8.7)	10 (11.4)	17 (8.0)	11 (9.5)	34 (15.7)	27 (23.1)				
	GG	4 (2.0)	1 (1.1)	1 (0.5)	0 (0.0)	17 (7.9)	3 (2.6)				
								<0.001	<0.001	0.02	0.001
	A	367 (93.6)	164 (93.2)	407 (95.5)	221 (95.3)	364 (84.3)	201 (85.9)				
	G	25 (6.4)	12 (6.8)	19 (4.5)	11 (4.7)	68 (15.7)	33 (14.1)				
PROCR rs867186								0.537	0.177	0.951	0.726
	AA	154 (78.6)	67 (76.1)	161 (75.6)	86 (74.1)	179 (82.9)	91 (77.8)				
	AG	40 (20.4)	20 (22.7)	49 (23.0)	28 (24.1)	35 (16.2)	25 (21.4)				
	GG	2 (1.0)	1 (1.1)	3 (1.4)	2 (1.7)	2 (0.9)	1 (0.9)				
								0.295	0.069	0.766	0.464
	A	348 (88.8)	154 (87.5)	371 (87.1)	200 (86.2)	393 (91.0)	207 (88.5)				
	G	44 (11.2)	22 (12.5)	55 (12.9)	32 (13.8)	39 (9.0)	27 (11.5)				
TRAF2 rs10781522								0.293	0.358	0.921	0.57
	AA	100 (51.0)	40 (45.5)	83 (39.0)	60 (51.7)	94 (43.5)	53 (45.3)				
	AG	79 (40.3)	39 (44.3)	111 (52.1)	42 (36.2)	98 (45.4)	50 (42.7)				
	GG	17 (8.7)	9 (10.2)	19 (8.9)	14 (12.1)	24 (11.1)	14 (12.0)				
								0.125	0.771	0.84	0.464
	A	279 (71.2)	119 (67.6)	277 (65.0)	162 (69.8)	286 (66.2)	156 (66.7)				
	G	113 (28.8)	57 (32.4)	149 (35.0)	70 (30.2)	146 (33.8)	78 (33.3)				

Exudative AMD males have a statistically significant difference in the distribution of *EXOC3L1* rs868213 AA, AG, and GG genotypes when compared to healthy men (90.5%, 9.5%, and 0.0% vs. 74.4%, 23.1%, and 2.6%, *p* = 0.003). However, males with early and exudative AMD had statistically substantially lower frequencies of the G allele of rs868213 than did the males in the control group (6.8% vs. 14.1%, *p* = 0.020; 4.7% vs. 14.1%, *p* = 0.001, respectively) (Table 7).

Analysis of *CXCL8* rs2227306 showed that the CT genotype increases the possibility of early AMD occurrence in females by 1.8-fold under the codominant model (OR = 1.807, 95% CI: 1.121–2.911, *p* = 0.015), while under the dominant model, the TT + CT genotypes increases these odds by 1.6-fold (OR = 1.606, 95% CI: 1.019–2.530, *p* = 0.041), but these results did not reach Bonferroni corrected significance level.

*EXOC3L1* rs868213 analysis revealed that the AG genotype decreases the odds of early AMD in females by 2 times (OR = 0.508, 95% CI: 0.274–0.943, *p* = 0.032) under the overdominant model. Under the recessive model, the GG genotype decreases these odd by 4.1-fold (OR = 0.244, 95% CI: 0.081–0.738, *p* = 0.012), when under the codominant model, AG and GG genotypes decreases these odds by 2.1-fold and 4.5-fold, respectively (OR = 0.471, 95% CI: 0.254–0.876, *p* = 0.017 and OR = 0.222, 95% CI: 0.073–0.673, *p* = 0.008, respectively). Unfortunately, these results did not survive strict Bonferroni correction. *EXOC3L1* rs868213 GG + AG genotypes compared with the AA genotype decrease the possibility of early AMD in females by 2.6-fold (OR = 0.388, 95% CI: 0.224–0.674, *p* < 0.001) under the dominant model. Also, each G allele decreased these odds by 2.1-fold under the additive model (OR = 0.471, 95% CI: 0.308–0.722, *p* < 0.001) (Table 8).

**Table 8:** Binomial logistic regression analysis of *CXCL8*, *MAP3K7*, *TNF/LTA*, *EXOC3L1*, *PROCR*, and *TRAF2* in the control and patients with early AMD females.

Model	Genotype/allele	OR (95% CI)	*p*-value	AIC
*CXCL8* rs2227306				
Codominant	CT vs. CC TT vs. CC	1.807 (1.121–2.911) 1.204 (0.678–2.141)	0.015 0.526	567.341
Dominant	TT + CT vs. CC	1.606 (1.019–2.530)	0.041	567.945
Recessive	TT vs. CC + CT	0.804 (0.502–1.287)	0.363	571.350
Overdominant	CT vs. CC + TT	1.655 (1.120–2.447)	0.363	565.743
Additive	T	1.114 (0.839–1.479)	0.455	571.622
*MAP3K7* rs157432				
Codominant	CT vs. CC TT vs. CC	0.887 (0.585–1.344) 1.518 (0.649–3.547)	0.572 0.335	572.678
Dominant	TT + CT vs. CC	0.959 (0.646–1.424)	0.837	572.140
Recessive	TT vs. CT + CC	1.585 (0.687–3.655)	0.280	570.998
Overdominant	CT vs. CC + TT	0.855 (0.568–1.287)	0.453	571.619
Additive	T	1.043 (0.757–1.436)	0.798	572.117
*TNF/LTA* rs2229094				
Codominant	TC vs. TT CC vs. TT	0.810 (0.537–1.222) 1.707 (0.683–4.265)	0.315 0.252	571.341
Dominant	CC + TC vs. TT	0.889 (0.599–1.318)	0.558	571.838
Recessive	CC vs. TC + TT	1.847 (0.749–4.556)	0.183	570.352
Overdominant	TC vs. TT + CC	0.777 (0.518–1.165)	0.222	570.687
Additive	C	1.002 (0.723–1.390)	0.988	572.182
*EXOC3L1* rs868213				
Codominant	AG vs. AA GG vs. AA	0.471 (0.254–0.876) 0.222 (0.073–0.673)	0.017 0.008	560.421
Dominant	GG + AG vs. AA	0.388 (0.224–0.674)	**< 0.001**	559.970
Recessive	GG vs. AA + AG	0.244 (0.081–0.738)	0.012	564.366
Overdominant	AG vs. AA + GG	0.508 (0.274–0.943)	0.032	567.352
Additive	G	0.471 (0.308–0.722)	**< 0.001**	558.421
*PROCR* rs867186				
Codominant	AG vs. AA GG vs. AA	1.328 (0.804–2.195) 1.162 (0.162–8.349)	0.268 0.881	572.941
Dominant	GG + AG vs. AA	1.319 (0.807–2.157)	0.269	570.958
Recessive	GG vs. AA + AG	1.103 (0.154–7.907)	0.922	572.172
Overdominant	AG vs. AA + GG	1.326 (0.803–2.190)	0.270	570.963
Additive	G	1.276 (0.808–2.014)	0.295	571.083
*TRAF2* rs10781522				
Codominant	AG vs. AA GG vs. AA	0.758 (0.503–1.140) 0.666 (0.337–1.317)	0.184 0.243	571.723
Dominant	GG + AG vs. AA	0.740 (0.502–1.091)	0.128	569.859
Recessive	GG vs. AA + AG	0.760 (0.395–1.461)	0.410	571.497
Overdominant	AG vs. AA + GG	0.813 (0.550–1.203)	0.300	571.106
Additive	G	0.793 (0.590–1.067)	0.126	569.826

OR - odds ratio; CI - confidence interval; *p* - significance level; AIC - Akaike information criteria.

A binary logistic regression analysis indicated that the *EXOC3L1* rs868213 AG and GG genotypes, were significantly associated with a decreased odds ratio of exudative AMD occurrence in females under the codominant genetic model. Specifically, the odds ratios were 2.4-fold, and 14.9-fold, respectively (OR = 0.418; CI: 0.215–0.814; *p* = 0.010, and OR = 0.067; CI: 0.008–0.531; *p* = 0.011,respectively). The AG genotype of *EXOC3L1* rs868213 lowers these odds by 2.2-fold under the overdominant model (OR = 0.450, 95% CI: 0.231–0.876, *p* = 0.019), while under the recessive model, GG decreases these odds by 13.5-fold (OR = 0.074, 95% CI: 0.009–0.588, *p* = 0.014). Unfortunately, these results did not survive strict Bonferroni correction. Also, the GG + AG genotypes are likely to be associated with 3.2-fold decreased odds of exudative AMD occurrence in women under the dominant model (OR = 0.317, 95% CI: 0.171–0.589, *p* < 0.001). Each G allele decreases the odds of developing exudative AMD in females by 2.8-folds (OR = 0.354; CI: 0.208–0.603; *p* < 0.001) (Table 9).

**Table 9:** Binomial logistic regression analysis of *CXCL8*, *MAP3K7*, *TNF/LTA*, *EXOC3L1*, *PROCR*, and *TRAF2* in the control and patients with exudative AMD females.

Model	Genotype/allele	OR (95% CI)	*p*-value	AIC
*CXCL8* rs2227306				
Codominant	CT vs. CC TT vs. CC	1.518 (0.921–2.500.921.500) 1.463 (0.818–2.614)	0.101 0.199	535.561
Dominant	TT + CT vs. CC	1.500 (0.936–2.403)	0.092	533.582
Recessive	TT vs. CT + CC	1.107 (0.690–1.779)	0.673	536.264
Overdominant	CT vs. CC + TT	1.258 (0.837–1.891)	0.269	535.218
Additive	T	1.212 (0.907–1.619)	0.195	534.751
*MAP3K7* rs157432				
Codominant	CT vs. CC TT vs. CC	1.136 (0.742–1.738) 0.965 (0.351–2.652)	0.557 0.945	538.069
Dominant	TT + CT vs. CC	1.117 (0.740–1.686)	0.598	536.165
Recessive	TT vs. CT + CC	0.917 (0.339–2.486)	0.865	536.414
Overdominant	CT vs. CC + TT	1.139 (0.749–1.732)	0.543	536.073
Additive	T	1.072 (0.755–1.524)	0.696	536.290
*TNF/LTA* rs2229094				
Codominant	TC vs. TT CC vs. TT	1.129 (0.739–1.726) 2.229 (0.851–5.841)	0.573 0.103	535.600
Dominant	CC + TC vs. TT	1.221 (0.811–1.839)	0.338	535.524
Recessive	CC vs. TC + TT	2.120 (0.822–5.466)	0.120	533.917
Overdominant	TC vs. TT + CC	1.054 (0.696–1.598)	0.803	536.380
Additive	C	1.274 (0.905–1.795)	0.165	534.503
*EXOC3L1* rs868213				
Codominant	AG vs. AA GG vs. AA	0.418 (0.215–0.814) 0.067 (0.008–0.531)	0.010 0.011	519.922
Dominant	GG + AG vs. AA	0.317 (0.171–0.589)	**< 0.001**	521.988
Recessive	GG vs. AA + AG	0.074 (0.009–0.588)	0.014	524.819
Overdominant	AG vs. AA + GG	0.450 (0.231–0.876)	0.019	530.683
Additive	G	0.354 (0.208–0.603)	**< 0.001**	518.605
*PROCR* rs867186				
Codominant	AG vs. AA GG vs. AA	1.416 (0.845–2.372) 2.055 (0.321–13.156)	0.186 0.447	536.194
Dominant	GG + AG vs. AA	1.449 (0.876–2.397)	0.149	534.344
Recessive	GG vs. AA + AG	1.921 (0.301–12.274)	0.490	535.954
Overdominant	AG vs. AA + GG	1.400 (0.837–2.344)	0.200	534.788
Additive	G	1.420 (0.896–2.251)	0.136	534.195
*TRAF2* rs10781522				
Codominant	AG vs. AA GG vs. AA	1.288 (0.838–1.979) 1.000 (0.480–2.084)	0.249 1.000	536.959
Dominant	GG + AG vs. AA	1.236 (0.817–1.870)	0.315	535.431
Recessive	GG vs. AA + AG	0.871 (0.435–1.747)	0.698	536.292
Overdominant	AG vs. AA + GG	1.288 (0.857–1.935)	0.224	534.959
Additive	G	1.102 (0.801–1.517)	0.550	536.085

OR - odds ratio; CI - confidence interval; *p* - significance level; AIC - Akaike information criteria.

We found that the *EXOC3L1* rs868213 AG genotype is associated with decreased odds by 2.4-fold of early AMD in males under the codominant genetic model (OR = 0.418; 95% CI: 0.190–0.920; *p* = 0.030). Also, GG + AG genotypes and AG are associated with decreased odds by 2.4-fold and 2.3-fold in early AMD in males under the dominant and overdominant genetic models (OR = 0.414; 95% CI: 0.195–0.882; *p* = 0.022, OR = 0.427; 95% CI: 0.195–0.938; *p* = 0.034, respectively). Further analysis showed that each G allele decreased early AMD odds in males under the additive model by 2.2-fold (OR = 0.465; 95% CI: 0.235–0.922; *p* = 0.028) (Table 10) Although these results did not survive after applying Bonferroni correction.

**Table 10:** Binomial logistic regression analysis of *CXCL8*, *MAP3K7*, *TNF/LTA*, *EXOC3L1*, *PROCR*, and *TRAF2* in the control and patients with early AMD males.

Model	Genotype/allele	OR (95% CI)	*p*-value	AIC
*CXCL8* rs2227306				
Codominant	CT vs. CC TT vs. CC	0.880 (0.464–1.669) 1.592 (0.744–3.406)	0.695 0.231	281.393
Dominant	TT + CT vs. CC	1.071 (0.593–1.934)	0.819	282.022
Recessive	CT vs. CC + TT	1.714 (0.881–3.335)	0.112	279.547
Overdominant	TT vs. CT + CC	0.729 (0.417–1.274)	0.267	280.835
Additive	T	1.225 (0.838–1.790)	0.294	280.970
*MAP3K7* rs157432				
Codominant	CT vs. CC TT vs. CC	0.923 (0.516–1.651) 0.354 (0.071–1.777)	0.788 0.207	282.230
Dominant	TT + CT vs. CC	0.844 (0.480–1.483)	0.555	281.725
Recessive	CT vs. CC + TT	0.365 (0.074–1.804)	0.217	280.302
Overdominant	TT vs. CT + CC	0.983 (0.554–1.746)	0.954	282.071
Additive	T	0.790 (0.487–1.282)	0.340	281.155
*TNF/LTA* rs2229094				
Codominant	TC vs. TT CC vs. TT	1.183 (0.659–2.122) 2.204 (0.591–8.219)	0.574 0.239	282.522
Dominant	CC + TC vs. TT	1.273 (0.726–2.233)	0.399	281.363
Recessive	TC vs. TT + CC	2.067 (0.565–7.560)	0.272	280.838
Overdominant	CC vs. TC + TT	1.112 (0.626–1.976)	0.717	281.943
Additive	C	1.305 (0.816–2.088)	0.266	280.834
*EXOC3L1* rs868213				
Codominant	AG vs. AA GG vs. AA	0.418 (0.190–0.920) 0.377 (0.038–3.696)	0.030 0.402	278.422
Dominant	GG + AG vs. AA	0.414 (0.195–0.882)	0.022	276.430
Recessive	GG vs. AA + AG	0.437 (0.045–4.272)	0.476	281.506
Overdominant	AG vs. AA + GG	0.427 (0.195–0.938)	0.034	277.221
Additive	G	0.465 (0.235–0.922)	0.028	276.710
*PROCR* rs867186				
Codominant	AG vs. AA GG vs. AA	1.087 (0.558–2.118) 1.358 (0.083–22.107)	0.807 0.830	283.974
Dominant	GG + AG vs. AA	1.097 (0.569–2.114)	0.782	281.998
Recessive	GG vs. AA + AG	1.333 (0.082–21.615)	0.840	282.033
Overdominant	AG vs. AA + GG	1.082 (0.556–2.107)	0.816	282.020
Additive	G	1.100 (0.595–2.032)	0.761	281.982
*TRAF2* rs10781522				
Codominant	AG vs. AA GG vs. AA	1.033 (0.575–1.858) 0.852 (0.335–2.164)	0.912 0.736	283.908
Dominant	GG + AG vs. AA	0.994 (0.570–1.732)	0.982	282.074
Recessive	GG vs. AA + AG	0.838 (0.345–2.035)	0.697	281.921
Overdominant	AG vs. AA + GG	1.067 (0.611–1.863)	0.821	282.023
Additive	G	0.959 (0.634–1.449)	0.841	282.034

OR - odds ratio; CI - confidence interval; *p* - significance level; AIC - Akaike information criteria.

The logistic regression analysis in exudative AMD males revealed that the *EXOC3L1* rs868213 AG genotype is associated with a 2.9-fold decreased odds of exudative AMD in males under the codominant genetic model (OR = 0.348; 95% CI: 0.162–0.743; *p* = 0.006), while the GG + AG genotypes decreased these odds by 3.1-fold under the over-dominant genetic model(OR = 0.320; 95% CI: 0.151–0.680; *p* = 0.003). Also, analysis showed that each G allele decreased by 3.1-fold exudative AMD odds in males under the additive model (OR = 0.324; 95% CI: 0.157–0.672; *p* = 0.002). Moreover, AG genotype decreased these odds by 2.8-fold under over-dominant genetic model (OR = 0.358; 95% CI: 0.167–0.765; *p* = 0.008), but this result did not reach Bonferroni corrected significance level. (Table 11).

**Table 11:** Binomial logistic regression analysis of *CXCL8*, *MAP3K7*, *TNF/LTA*, *EXOC3L1*, *PROCR*, and *TRAF2* in the control and patients with exudative AMD males.

Model	Genotype/allele	OR (95% CI)	*p*-value	AIC
*CXCL8* rs2227306				
Codominant	CT vs. CC TT vs. CC	1.508 (0.820–2.772) 1.660 (0.774–3.559)	0.186 0.193	318.983
Dominant	TT + CT vs. CC	1.549 (0.870–2.760)	0.137	317.057
Recessive	CT vs. CC + TT	1.278 (0.664–2.461)	0.463	318.748
Overdominant	TT vs. CT + CC	1.228 (0.729–2.069)	0.441	318.693
Additive	T	1.309 (0.897–1.910)	0.163	317.325
*MAP3K7* rs157432				
Codominant	CT vs. CC TT vs. CC	0.960 (0.553–1.665) 0.787 (0.249–2.489)	0.883 0.684	321.118
Dominant	TT + CT vs. CC	0.935 (0.551–1.587)	0.803	319.227
Recessive	CT vs. CC + TT	0.801 (0.259–2.476)	0.700	319.140
Overdominant	TT vs. CT + CC	0.981 (0.572–1.684)	0.945	319.285
Additive	T	0.925 (0.599–1.428)	0.724	319.164
*TNF/LTA* rs2229094				
Codominant	TC vs. TT CC vs. TT	0.984 (0.566–1.711) 1.235 (0.316–4.826)	0.955 0.762	321.187
Dominant	CC + TC vs. TT	1.007 (0.590–1.719)	0.979	319.289
Recessive	TC vs. TT + CC	1.242 (0.322–4.784)	0.753	319.190
Overdominant	CC vs. TC + TT	0.972 (0.563–1.680)	0.919	319.279
Additive	C	1.031 (0.652–1.629)	0.896	319.272
*EXOC3L1* rs868213				
Codominant	AG vs. AA GG vs. AA	0.348 (0.162–0.743) -	**0.006** **-**	310.169
Dominant	GG + AG vs. AA	0.320 (0.151–0.680)	**0.003**	309.640
Recessive	GG vs. AA + AG	-	**-**	-
Overdominant	AG vs. AA + GG	0.358 (0.167–0.765)	0.008	311.687
Additive	G	0.324 (0.157–0.672)	**0.002**	308.715
*PROCR* rs867186				
Codominant	AG vs. AA GG vs. AA	1.184 (0.637–2.201) 1.875 (0.166–21.231)	0.593 0.612	320.765
Dominant	GG + AG vs. AA	1.212 (0.660–2.226)	0.535	318.903
Recessive	GG vs. AA + AG	1.802 (0.160–20.319.160.319)	0.634	319.050
Overdominant	AG vs. AA + GG	1.172 (0.631–2.176)	0.615	319.037
Additive	G	1.219 (0.695–2.138)	0.491	318.812
*TRAF2* rs10781522				
Codominant	AG vs. AA GG vs. AA	0.764 (0.436–1.339) 0.861 (0.373–1.987)	0.347 0.726	320.399
Dominant	GG + AG vs. AA	0.786 (0.467–1.324)	0.366	318.472
Recessive	GG vs. AA + AG	0.968 (0.435–2.155)	0.937	319.283
Overdominant	AG vs. AA + GG	0.787 (0.461–1.345)	0.381	318.522
Additive	G	0.875 (0.599–1.277)	0.489	318.809

OR - odds ratio; CI - confidence interval; *p* - significance level; AIC - Akaike information criteria.

### Treatment efficiency

The response to treatment was assessed in 106 exudative AMD patients. Table 12 lists the clinical and demographic features of the population. There was no difference in the percentage of respondents and non-respondents by gender or age.

**Table 12:** The treatment efficiency parameters.

Characteristic		Non-responders *n* = 20	Responders *n* = 86	*p*-value
Gender	Females, n (%)	12 (60.0)	59 (68.6)	0.461*
	Males, n (%)	8 (40.0)	27 (31.4)	
Age years; mean (SD)		75.60 (6.75)	76.90 (8.29)	0.495**
**Response parameter**				
VA, median (IQR)				
Before treatment		0.29 (0.34)^1^	0.35(0.27)^2^	0.257**
After 3 months		0.21 (0.35)^1^	0.30 (0.33)^2^	0.282***
After 6 months		0.21 (0.28)^1^	0.35 (0.32)^2^	0.072***
CRT (µm), median (IQR)				
Before treatment		456 (203.5)^3^	300 (101)^4^	0.001***
After 3 months		292 (127)^3^	266 (84)^4^	0.357***
After 6 months		284 (107.5)^3^	278 (97)^4^	0.531***

*p* - significance level, significant when *p* < 0.05; IQR - interquartile range; SD - standard deviation; VA - visual acuity; CRT - central retinal thickness; * Pearson’s chi-squared test; ** Student’s *t* test; *** Mann-Whitney U test; 1Friedman test, *p* = 0.741 × 2 = 0.600 df = 2; 2Friedman test, *p* = 0.071 × 2 = 5.299 df = 2; 3Friedman test, *p* = 0.005 × 2 = 10.706 df = 2; 4Friedman test, *p* < 0.001 × 2 = 18.578 df = 2.

We determined that the central retinal thickness (CRT) was lower in responders compared to the non-responders before the treatment (300 (101) vs. 456 (203.5), *p* < 0.001) (Table 13).

**Table 13:** Associations between *CXCL8*, *MAP3K7*, *TNF/LTA*, *EXOC3L1*, *PROCR*, and *TRAF2* SNVs and response to treatment.

Genetic model	Genotype/Allele	Non-responders *n* = 20	Responders *n* = 86	OR (95% CI)	*p*-value	AIC
*CXCL8* rs2227306						
Codominant	CT vs. CC TT vs. CC	12 (60.0) 5 (25.0)	44 (51.2) 16 (18.6)	0.423 (0.109–1.640) 0.369 (0.078–1.759)	0.213 0.211	104.536
Dominant	TT + CT vs. CC	17 (85.0)	60 (69.8)	0.407 (0.110–1.511)	0.179	102.586
Recessive	CT vs. CC + TT	5 (25.0)	16 (18.6)	0.686 (0.217–2.163)	0.520	104.273
Overdominant	TT vs. CT + CC	12 (60.0)	44 (51.2)	0.698 (0.260–1.879)	0.477	104.160
Additive	T	22 (55.0)	76 (44.2)	0.627 (0.304–1.292)	0.206	103.040
*MAP3K7* rs157432						
Codominant	CT vs. CC TT vs. CC	5 (25) 1 (5.0)	30 (34.9) 1 (1.2)	1.527 (0.501–4.652) 0.255 (0.015–4.327)	0.456 0.344	105.087
Dominant	TT + CT vs. CC	6 (30.0)	31 (36.0)	1.315 (0.459–3.769)	0.610	104.406
Recessive	CT vs. CC + TT	1 (5.0)	1 (1.2)	0.224 (0.013–3.735)	0.297	103.667
Overdominant	TT vs. CT + CC	5 (25.0)	30 (34.9)	1.607 (0.952–4.852)	0.400	103.928
Additive	T	7 (17.5)	32 (18.6)	1.086 (0.421–2.804)	0.864	104.643
*TNF/LTA* rs2229094						
Codominant	TC vs. TT CC vs. TT	8 (40.0) 2 (10.0)	28 (32.6) 6 (7.0)	0.673 (0.239–1.899) 0.577 (0.102–3.279)	0.454 0.535	105.920
Dominant	CC + TC vs. TT	10 (50.0)	34 (39.5)	0.654 (0.246–1.737)	0.394	103.948
Recessive	TC vs. TT + CC	2 (10.0)	6 (7.0)	0.675 (0.126–3.622)	0.647	104.473
Overdominant	CC vs. TC + TT	8 (40.0)	28 (32.6)	0.724 (0.266–1.972)	0.528	104.279
Additive	C	12 (30.0)	40 (23.3)	0.726 (0.348–1.514)	0.393	103.961
*EXOC3L1* rs868213						
Codominant	AG vs. AA GG vs. AA	1 (5.0) -	8 (9.3) -	1.949 (0.230–16.538.230.538) -	0.541 -	106.238
Dominant	GG + AG vs. AA	1 (5.0)	8 (9.3)	1.949 (0.230–16.538.230.538)	0.541	104.238
Recessive	GG vs. AA + AG	-	-	-	-	-
Overdominant	AG vs. AA + GG	1 (5.0)	8 (9.3)	1.949 (0.230–16.538.230.538)	0.541	104.238
Additive	G	1 (5.0)	8 (9.3)	1.949 (0.230–16.538.230.538)	0.541	104.238
*PROCR* rs867186						
Codominant	AG vs. AA GG vs. AA	6 (30.0) 0 (0.0)	27 (31.4) 2 (2.3)	1.105 (0.383–3.191) -	0.853 -	105.792
Dominant	GG + AG vs. AA	6 (30.0)	29 (33.7)	1.187 (0.413–3.412)	0.750	104.569
Recessive	GG vs. AA + AG	-	2 (2.3)	-	-	-
Overdominant	AG vs. AA + GG	6 (30.0)	27 (31.4)	1.068 (0.370–3.080)	0.903	104.657
Additive	G	6 (30.0)	31 (18.0)	1.269 (0.472–3.409)	0.637	104.442
*TRAF2* rs10781522						
Codominant	AG vs. AA GG vs. AA	6 (30.0) 3 (15.0)	35 (40.7) 11 (12.8)	1.604 (0.538–4.787) 1.008 (0.239–4.258)	0.397 0.991	105.868
Dominant	GG + AG vs. AA	9 (45.0)	46 (53.5)	1.406 (0.529–3.736)	0.495	104.204
Recessive	GG vs. AA + AG	3 (15.0)	11 (12.8)	0.831 (0.209–3.307)	0.793	104.605
Overdominant	AG vs. AA + GG	6 (30.0)	35 (40.7)	1.601 (0.561–4.570)	0.379	103.868
Additive	G	12 (30.0)	57 (33.1)	1.139 (0.561–2.312)	0.718	104.540

However, the VA and CRT parameters before and after therapy were compared using a Friedman test. The Friedman test did not reveal a significant difference in VA across the three measurement time points in the non-responder group (χ² (2) = 0.600, *p* = 0.741) and responder group (χ² (2) = 5.299, *p* = 0.071). While post-hoc pairwise analysis comparisons with Wilcoxon signed-rank tests indicated that VA was significantly higher after the 6 months of treatment compared with baseline (0.35 (0.32) vs. 0.35 (0.27), *p* = 0.047) in the responder group (Table 12).

The Friedman test revealed that the CRT parameter was also decreased after the treatment in the non-responders’ and responders’ groups, respectively (χ² (2) = 10.706, *p* = 0.005 and χ² (2) = 18.578, *p* < 0.001). Post-hoc pairwise analysis comparisons with Wilcoxon signed-rank tests indicated that CRT was significantly thinner after the 3 months of treatment compared with baseline (292 (127) vs. 456 (203.5), *p* < 0.001) in the non-responder group. Also, CRT was significantly thinner after 6 months of treatment compared with baseline (284 (107.5) vs. 456 (203.5), *p* = 0.003). Post-hoc pairwise analysis comparisons with Wilcoxon signed-rank tests indicated that CRT was significantly thinner after the 3 months of treatment compared with baseline (266 (84) vs. 300 (101), *p* < 0.001) in the responder group. Also, CRT was significantly thinner after 6 months of treatment compared with baseline (278 (97) vs. 300 (101), *p* < 0.001) (Table 12).

Binomial logistic regression analysis was used to examine the connection between all SNVs and the response to anti-VEGF injections. The analysis does not show any statistically significant results (Table 13).

### Elisa results

**Fig. 1:**
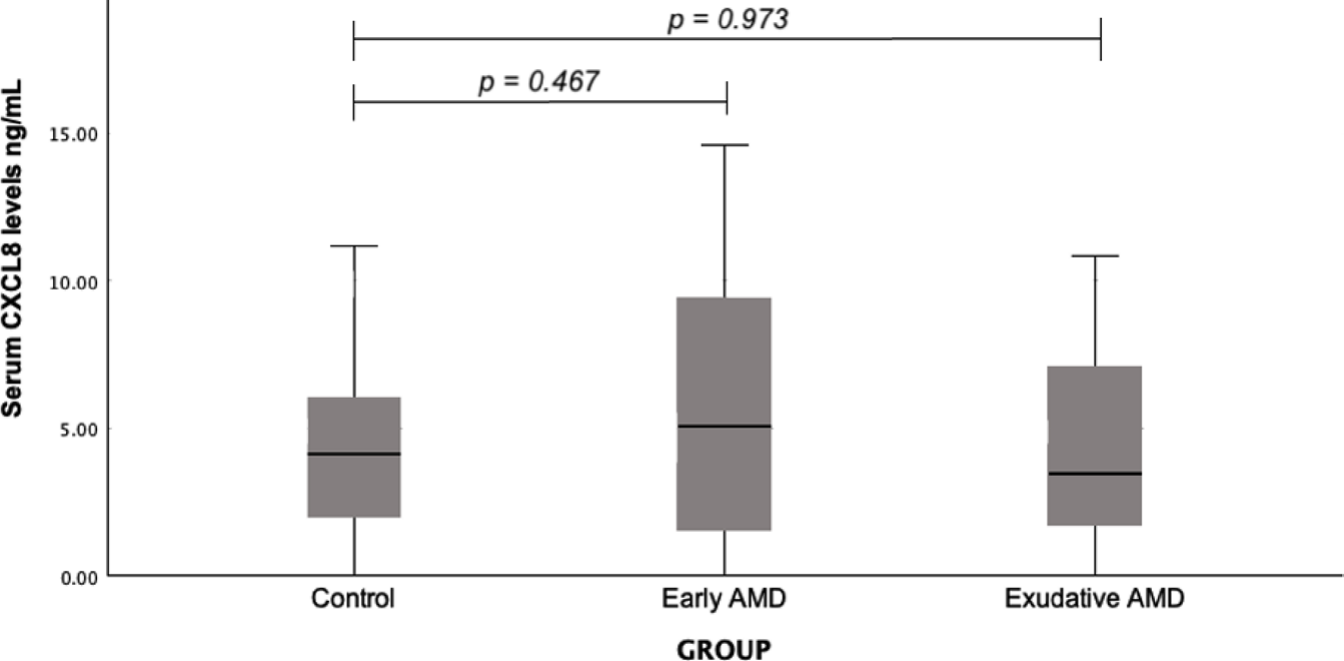
Serum CXCL8 levels in patients with early and exudative AMD and the control group; a Mann-Whitney U test was used.

Serum CXCL8 concentrations were assessed in patients with early and exudative AMD as well as in control subjects. A statistically significant deviation between patients who developed early AMD and the controls (median (IQR): 5.05 (8.13) vs. 4.11 (4.40), *p* = 0.467) or between patients with exudative AMD and controls was not found (median (IQR): 3.44 (5.59) vs. 4.11 (4.40), *p* = 0.973) (Fig. 1).

**Fig. 2:**
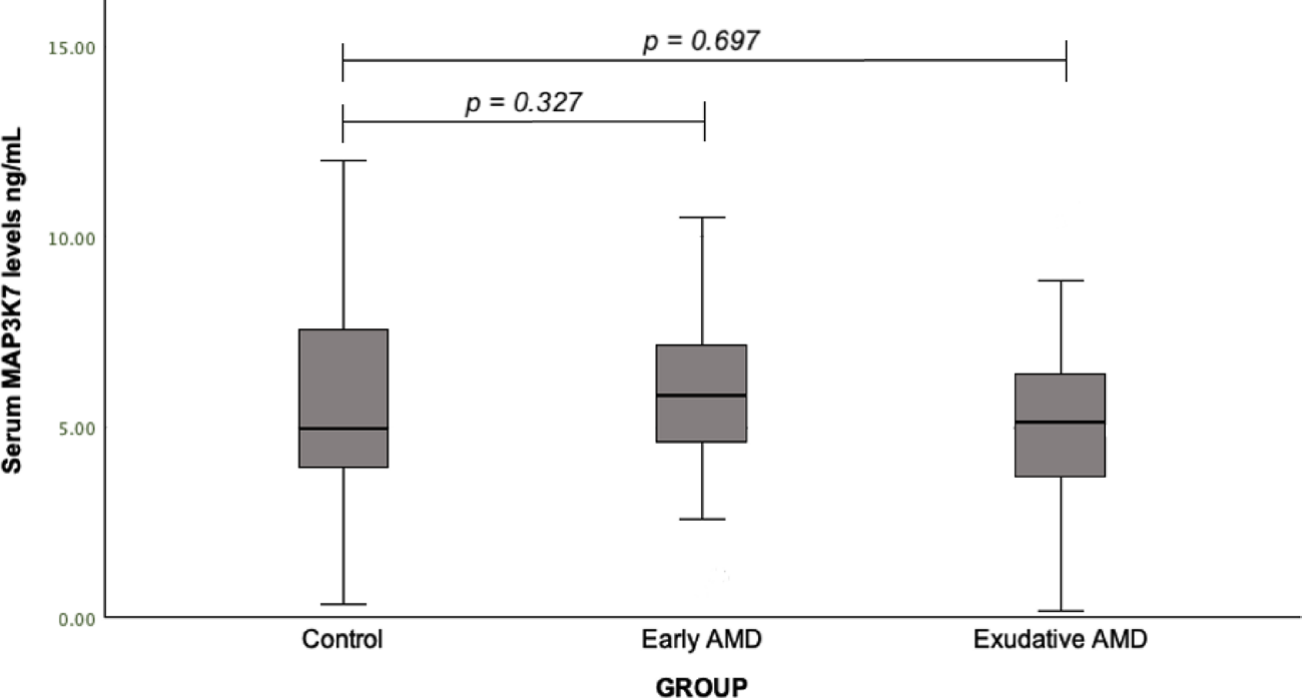
Serum MAP3K7 levels in patients with early and exudative AMD and the control group; a Mann-Whitney U test was used.

We evaluated MAP3K7 serum levels between early AMD and control group (median (IQR): 5.83 (3.06) vs. 4.96 (3.64), *p* = 0.327), and between exudative AMD and control group (median (IQR): 5.16 (2.89) vs. 4.96 (3.64), *p* = 0.697), but no statistically significant differences were found (Fig. 2).

**Fig. 3:**
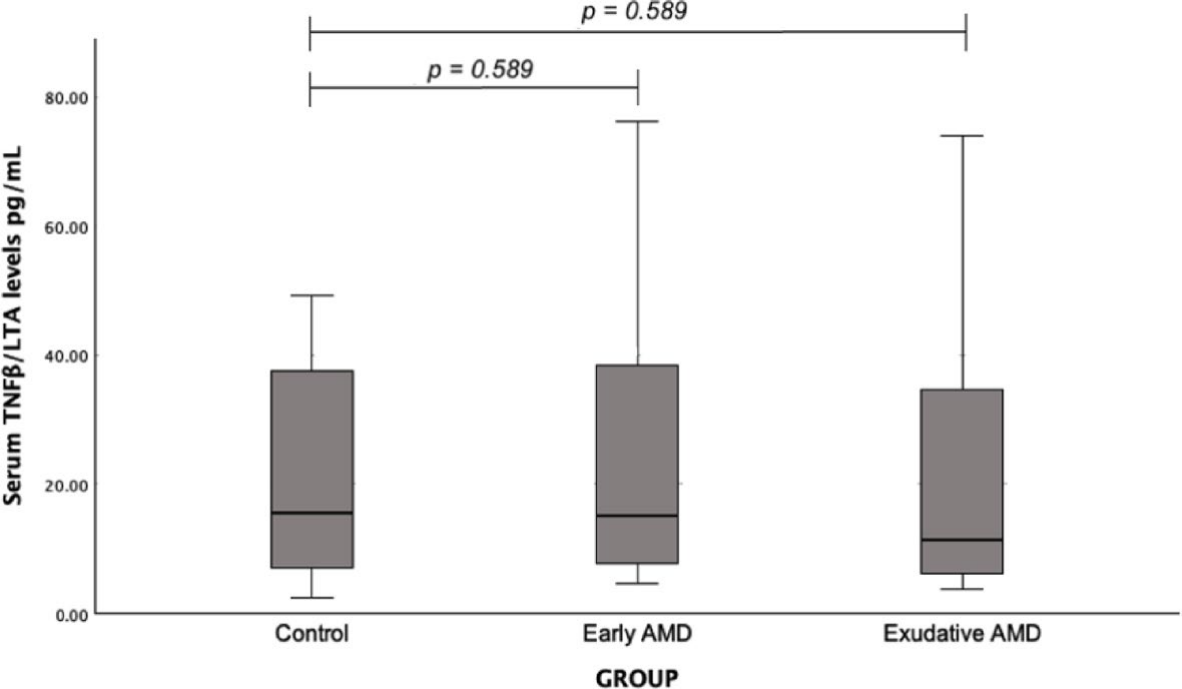
Serum TNF/LTA levels in patients with early and exudative AMD and the control group; a Mann-Whitney U test was used.

The concentration of TNF/LTA in the blood serum was examined in the groups of early and exudative AMD patients, and healthy individuals, but no statistically significant difference was found (median (IQR): 15.13 (35.16) vs. 15.64 (31.34), *p* = 0.589; median (IQR): 11.27 (31.44) vs. 15.64 (31.34), *p* = 0.589, respectively) (Fig. 3).

**Fig. 4:**
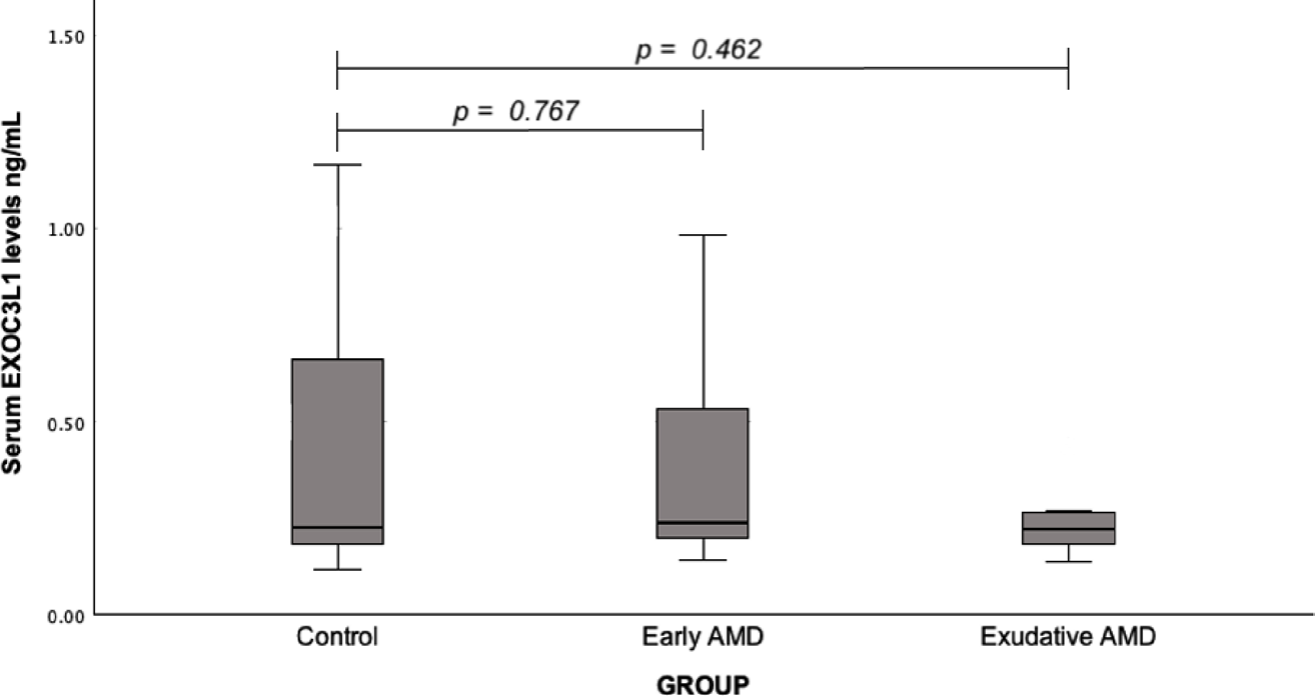
Serum EXOC3L1 levels in patients with early and exudative AMD and the control group; a Mann-Whitney U test was used.

The EXOC3L1 serum levels were also measured between early AMD and control group (median (IQR): 0.24 (0.42) vs. 0.23 (0.54), *p* = 0.767), and between exudative AMD and control group (median (IQR): 0.22 (0.08) vs. 0.23 (0.54), *p* = 0.462), but no statistically significant differences were found (Fig. 4).

**Fig. 5:**
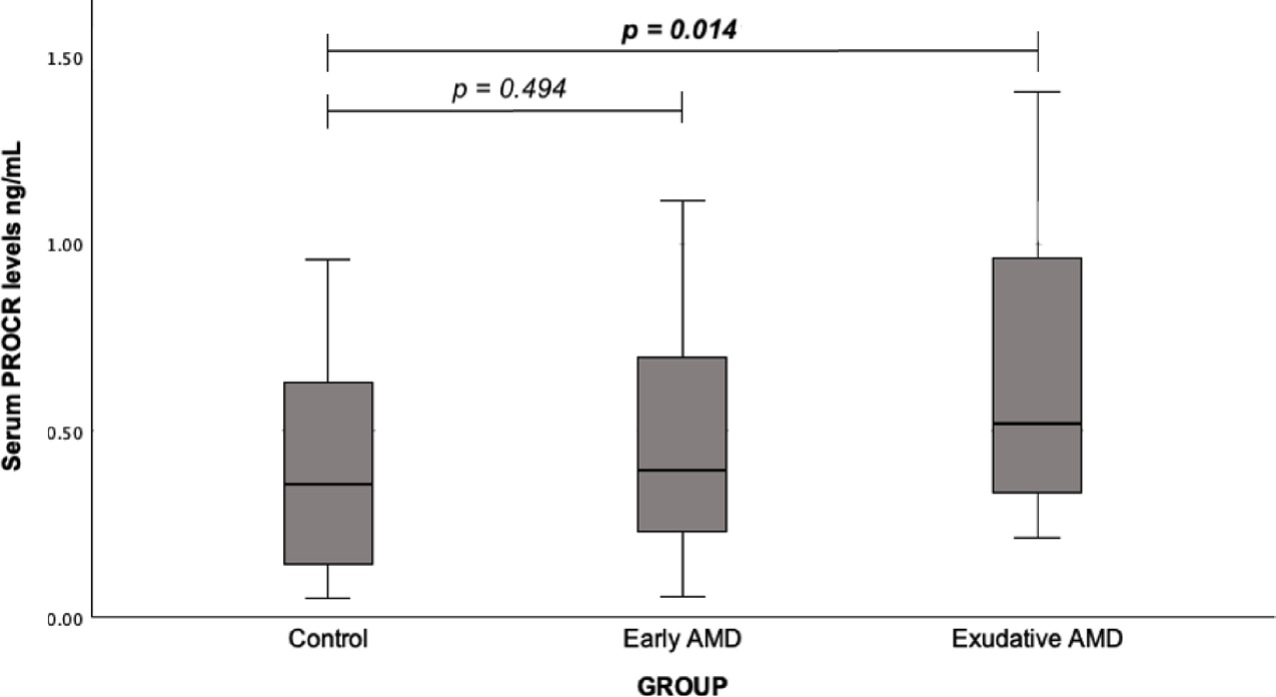
Serum PROCR levels in patients with early and exudative AMD and the control group; a Mann-Whitney U test was used.

The blood serum concentration of PROCR in the early and exudative AMD patient and healthy individual groups was evaluated. It was found that PROCR serum concentration was statistically significantly higher in exudative AMD patients compared with the control group (median (IQR): 0.52 (0.64) vs. 0.36 (0.51), *p* = 0.014). No statistically significant differences were found between early AMD patients and control groups (median (IQR): 0.40 (0.49) vs. 0.36 (0.51), *p* = 0.494) (Fig. 5).

**Fig. 6:**
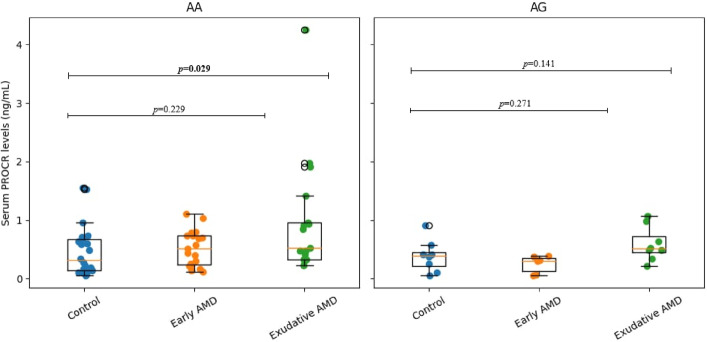
Serum PROCR levels across different PROCR rs867186 genotypes in the control, early, and exudative AMD study groups; a Mann-Whitney U test was used.

Since the results of PROCR were statistically significant, the serum levels of PROCR was analyzed across different genotypes. Patients who developed exudative AMD with the AA genotype of *PROCR* rs867186 had significantly increased serum PROCR concentrations compared to the controls (median (IQR): 0.52 (0.86) vs. 0.31 (0.56), *p* = 0.029). The GG genotype was not observed in the study cohort and therefore was not included in the analysis (Fig. 6).

**Fig. 7:**
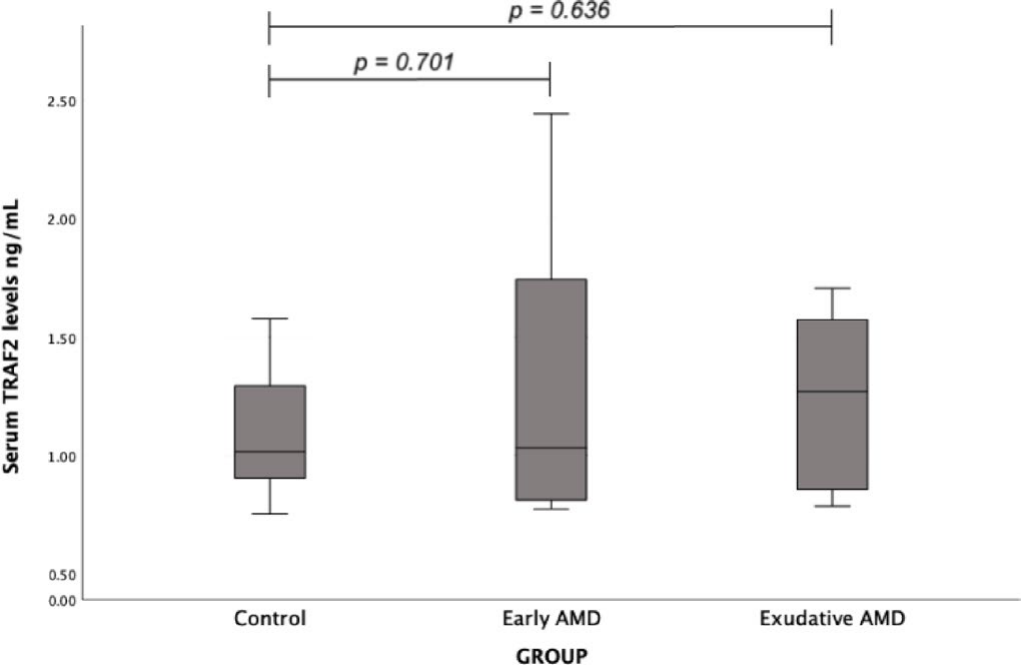
Serum TRAF2 levels in patients with early and exudative AMD and the control group; a Mann-Whitney U test was used.

The TRAF2 serum levels were determined between early AMD and control group (median (IQR): 1.03 (0.94) vs. 1.01 (0.40), *p* = 0.701), and between exudative AMD and control group (median (IQR): 1.27 (0.73) vs. 1.01 (0.40), *p* = 0.636), but no statistically significant differences were found (Fig. 7).

## Discussion

Age-related macular degeneration (AMD) is a multifactorial retinal disorder with a pronounced genetic component. Although complement system genes have been extensively investigated, increasing evidence indicates that inflammatory signaling, angiogenesis, oxidative stress responses, intracellular trafficking, and vascular regulation also contribute to AMD susceptibility and progression. In this study, we evaluated genetic variants and circulating biomarkers related to CXCL8 (IL-8), MAP3K7, LTA (TNF-β), EXOC3L1, PROCR, and TRAF2, representing both established and emerging pathways potentially involved in AMD pathophysiology.

Among the analyzed genes, CXCL8 (IL-8) demonstrated the most consistent association with AMD, particularly the exudative form. The rs2227306 (+ 781 C/T) variant has been repeatedly associated with increased AMD susceptibility, with Ambreen et al. reporting a higher frequency of the T allele and elevated serum IL-8 levels in AMD patients, supporting its functional relevance^33^. Similar associations were described by Ricci et al. through IL-8 haplotype analysis^34^, and these findings were further corroborated by meta-analyses demonstrating a robust association with neovascular AMD^35,36^. Consistent with this evidence, our results showed that *CXCL8* rs2227306 CT and TT + CT genotypes were associated with increased odds of exudative AMD, with additional sex-specific associations observed for early AMD in females. Functionally, IL-8 is known to promote VEGF-independent angiogenesis, particularly under oxidative stress conditions. Reactive oxygen species enhance IL-8 expression via MAPK signaling, thereby sustaining chronic inflammation and choroidal neovascularization—key processes in AMD progression.

MAP3K7, encoding transforming growth factor-β–activated kinase 1 (TAK1), represents a biologically compelling but genetically less established candidate. Functional studies indicate that TAK1 integrates inflammatory cytokine and oxidative stress signals through NF-κB and MAPK pathways. Experimental data demonstrate that TAK1 activation in endothelial and RPE cells promotes angiogenesis and regulates autophagy, cellular senescence, and extracellular matrix remodeling^37–39^. Despite this strong biological plausibility, our study did not identify significant associations between *MAP3K7* rs157432 or serum MAP3K7 levels and AMD. These findings highlight an important distinction between functional relevance and detectable genetic effects in population-based studies and suggest that MAP3K7 may influence AMD through downstream signaling or context-dependent mechanisms not captured by common variants.

The *LTA (TNF-β)* gene, encoding a cytokine involved in immune regulation and apoptosis, was evaluated due to the established role of TNF-family signaling in retinal inflammation and neovascularization. Although *LTA* variants such as rs2229094 have been implicated in systemic inflammatory and vascular diseases^40,41^, our analysis did not reveal significant associations with AMD or circulating TNF/LTA levels. These results suggest that while TNF-mediated inflammation is relevant to AMD pathogenesis, LTA itself may exert indirect or modest genetic effects, potentially influenced by linkage disequilibrium with neighboring immune-related loci.

EXOC3L1 emerged as a notable finding in this study, demonstrating a protective association against both early and exudative AMD. Although EXOC3L1 has not been previously linked to AMD, its role in vesicular trafficking, exocytosis, and cellular homeostasis provides strong biological plausibility. Proper exocyst function is essential for RPE physiology, autophagy, and photoreceptor outer segment clearance—processes disrupted in retinal degeneration. Supporting evidence from related EXOC3L family members implicates these genes in endothelial signaling, neurodevelopmental disorders, and neurodegenerative disease^42–47^. Our findings suggest that intracellular trafficking and stress-response mechanisms may represent underexplored contributors to AMD susceptibility and warrant further investigation.

The *PROCR* gene, encoding the endothelial protein C receptor (EPCR), was examined in the context of vascular stability and inflammation. Although *PROCR* variants have not been widely studied in AMD, experimental evidence indicates that EPCR signaling contributes to pathological retinal neovascularization^21^. The rs867186 (Ser219Gly) variant has been extensively associated with thromboinflammatory and cardiovascular disorders^48–50^. In our cohort, patients with exudative AMD carrying the AA genotype exhibited significantly increased serum PROCR levels, supporting a potential link between endothelial dysfunction and neovascular AMD progression.

Finally, TRAF2, a key adaptor protein mediating TNF receptor signaling and NF-κB activation, showed no significant genetic or biomarker associations with AMD in our study. Although *TRAF2* variants have been implicated in autoimmune and inflammatory diseases, including ankylosing spondylitis^51^, our findings suggest a limited role for TRAF2 in AMD, at least for the variants and biomarkers analyzed.

Collectively, these results do not support a single unifying pathogenic pathway but instead emphasize the heterogeneous molecular architecture of AMD, in which inflammatory, vascular, and intracellular trafficking mechanisms contribute in a stage- and context-dependent manner. Integrating genetic and biomarker data across multiple biological pathways may therefore enhance risk stratification and guide future mechanistic and translational studies in AMD.

Despite these findings, study has several limitations that should be acknowledged. The relatively small number of patients in the case group may have limited the statistical significance to detect significant associations, which could partly explain the lack of observed relationships. Therefore, larger studies with adequately powered sample sizes are required to validate these findings. Hardy–Weinberg equilibrium (HWE) was evaluated within each study group and one SNV deviated from HWE in the control and early AMD groups, but not in the exudative AMD group. The SNV was retained because (i) its minor allele frequency was comparable to that reported in public reference datasets for similar ancestry, (ii) standard genotyping quality checks did not indicate technical artifacts (e.g., high call rate and no evidence of differential missingness), and (iii) HWE deviation can arise from population substructure, cryptic relatedness, or ascertainment/selection effects, particularly in community-based controls and clinically heterogeneous early disease categories.

While age and sex were included as covariates in all analyses, adjustment for other established risk factors for age-related macular degeneration, such as smoking status, body mass index, cardiovascular comorbidities, and lifestyle-related factors, was not feasible due to incomplete data availability. As the primary aim of this study was to explore the role of genetic variants in disease development and treatment response, these factors were beyond the scope of the present analyses and should be considered in future studies.

## Conclusion

The genes analyzed in this study are involved in diverse biological pathways and were evaluated for their genetic and biomarker associations with AMD. While *CXCL8/IL-8 and MAP3K7* have been linked to retinal degeneration and angiogenesis in previous studies, the present work does not directly assess these processes. *LTA*,* EXOC3L1*, and *PROCR*represent emerging candidates whose roles in AMD require further investigation. Elucidating the contribution of these genes may support the future development of improved diagnostic approaches and targeted therapeutic strategies for AMD.

## Supplementary Information

Below is the link to the electronic supplementary material.

Supplementary Material 1

## Data Availability

The data from this study are not publicly available in order to protect participant privacy but can be provided upon reasonable request.
