# Supplementary material for: Genetic variants and serum biomarkers of CXCL8, MAP3K7, LTA/TNF, EXOC3L1, PROCR, and TRAF2 in Age-Related macular degeneration: associations with disease risk and therapeutic response
Source: Sci Rep. 2026 Mar 17;16:13793. doi: 10.1038/s41598-026-42838-9 (PMC13129072; doi:10.1038/s41598-026-42838-9)
Supplement: Supplementary file 1 — Supplementary Material 1 [file 41598_2026_42838_MOESM1_ESM.docx]

Supplementary Material.

**Table 1**. Additional information on the protein analysis

| ***Protein*** | ***Sensitivity*** | ***Range*** | ***Manufacturer*** |
| --- | --- | --- | --- |
| CXCL8 | < 5.0 pg/mL | 0 - 1000 pg/mL | Invitrogen IL-8 ELISA Kit (Human), Vienna, Austria |
| MAP3K7 | < 0.14 ng/mL | 0.312 ng/mL - 20 ng/mL | Human MAP3K7 ELISA Kit, Abbexa, Cambridge, UK |
| TNF/LTA | < 4.6 pg/mL | 1.0 - 1125 pg/mL | Invitrogen TNFα ELISA Kit (Human), Vienna, Austria |
| EXOC3L1 | < 0.06 ng/mL | 0.156 ng/mL - 10 ng/mL | Human EXOC3L1 ELISA Kit, Abbexa, Cambridge, UK |
| PROCR | < 0.26 ng/mL | 0.625 ng/mL - 40 ng/mL | Human PROCR ELISA Kit, Abbexa, Cambridge, UK |
| TRAF2 | < 0.38 ng/mL | 0.78 ng/mL - 50 ng/mL | Human TRAF2 ELISA Kit, Abbexa, Cambridge, UK |
